# Changes in physical activity 1 year after breast cancer diagnosis and associations with patient-reported outcomes: results from the AMBER cohort

**DOI:** 10.1093/abm/kaaf098

**Published:** 2025-12-05

**Authors:** Jeff Vallance, Christine Friedenreich, Ruixuan Zhang, Qinggang Wang, Charles Matthews, Lin Yang, Margaret McNeely, Chad Wagoner, Nicole Culos-Reed, Gordon Bell, Jessica McNeil, Leanne Dickau, Kerry Courneya

**Affiliations:** Faculty of Health Disciplines, Athabasca University, 1 University Dr, Athabasca, AB, T9S-3A3, Canada; Department of Cancer Epidemiology and Prevention Research, Alberta Health Services, Calgary, AB, T2N-5G2, Canada; Departments of Oncology and Community Health Sciences, Cumming School of Medicine, University of Calgary, Calgary, AB, T2N-5G2, Canada; Department of Cancer Epidemiology and Prevention Research, Alberta Health Services, Calgary, AB, T2N-5G2, Canada; Department of Cancer Epidemiology and Prevention Research, Alberta Health Services, Calgary, AB, T2N-5G2, Canada; Division of Cancer Epidemiology and Genetics, US National Cancer Institute, Bethesda, MD, 20850, United States; Department of Cancer Epidemiology and Prevention Research, Alberta Health Services, Calgary, AB, T2N-5G2, Canada; Departments of Oncology and Community Health Sciences, Cumming School of Medicine, University of Calgary, Calgary, AB, T2N-5G2, Canada; Faculty of Rehabilitation Medicine, College of Health Sciences, University of Alberta, Edmonton, AB, T6G-2G4, Canada; Department of Kinesiology, Recreation, and Sport Studies, University of Tennessee, Knoxville, TN, 37996-2700, United States; Faculty of Kinesiology, University of Calgary, Calgary, AB, T2N-1N4, Canada; Faculty of Kinesiology, Sport, and Recreation, College of Health Sciences, University of Alberta, Edmonton, AB, T6G-2H9, Canada; Department of Kinesiology, School of Health and Human Sciences, University of North Carolina at Greensboro, Greensboro, NC, 27412, United States; Department of Cancer Epidemiology and Prevention Research, Alberta Health Services, Calgary, AB, T2N-5G2, Canada; Faculty of Kinesiology, Sport, and Recreation, College of Health Sciences, University of Alberta, Edmonton, AB, T6G-2H9, Canada

**Keywords:** breast cancer, physical activity, walking, quality of life, fatigue, accelerometers, prospective cohort, survivorship

## Abstract

**Background:**

Few studies have prospectively examined how changes in physical activity in the first year after a breast cancer diagnosis may affect patient-reported outcomes (PROs) including quality of life and fatigue.

**Purpose:**

The purpose of this study was to examine changes in device-measured physical activity and associations with changes in quality of life [physical composite summary score (PCS) and mental composite summary score (MCS)] and fatigue from the first year after diagnosis among newly diagnosed women with breast cancer in the Alberta Moving Beyond Breast Cancer Study.

**Methods:**

In this prospective cohort study, we assessed women within a median of 60 days postsurgery (*N* = 1442) and again 1 year later (*N* = 1194). At both timepoints, participants wore an ActiGraph accelerometer for 7 days to measure light and moderate-to-vigorous physical activity (MVPA) and an activPAL accelerometer for daily steps. We used analysis of covariance to compare PRO change scores (dependent variables: PCS and MCS, and fatigue) across activity change quartiles (Q).

**Results:**

Participants were categorized into Q1 (decreased activity: mean changes = −37.2 minutes/day), Q2 (stable activity: mean change = −4.8 minutes/day), Q3 (modest increase in activity: mean change = 13.2 minutes/day), and Q4 (large increase in activity: mean change = +49.8 minutes/day). For MVPA, participants in Q4 had significantly larger improvements in PCS and MCS compared with those in the lowest quartiles (PCS: Q1 Δ = 1.5 points, *P* = .026; Q2 Δ = 1.6 points, *P* = .017; MCS: Q1 Δ = 2.2 points, *P* = .007). Significant differences also emerged for fatigue as participants in Q4 of MVPA reported improvements in fatigue compared to those in Q1 (Δ = 1.9 points, *P* = .017) and Q2 (Δ = 1.9 points, *P* = .016). Improvements in PCS, MCS, and fatigue were observed when comparing the highest quartile of change (Q4) in light intensity activity, daily steps, and MVPA in ≥10-minute bouts to those in Q1 and Q2.

**Conclusions:**

Women with breast cancer who increased physical activity from diagnosis to 1 year had significantly better improvements in PCS, MCS, and fatigue compared with those who decreased or maintained their physical activity.

## Introduction

The World Health Organization estimates 2.3 million women were diagnosed with breast cancer in 2022, and 670 000 died from this disease.^1^ Despite advances in treatments, patient-reported outcomes (PROs) including quality of life (QoL) and fatigue remain important considerations after a breast cancer diagnosis, and are important outcome indicators in women with breast cancer.^2^ After diagnosis, PROs are predictive of both adverse events and overall survival.^3^ In a recent pooled analysis of clinical trials, PROs were associated with clinical outcomes in a linear association, with physical functioning being the most prognostic for overall survival.^4^ For mental health, research suggests close to 40% of newly diagnosed breast cancer survivors report clinically significant symptoms.^5^ Positive associations between physical activity and PROs after individuals have completed adjuvant therapy for breast cancer are well established. Less is known about the changes in physical activity and PROs during treatment and the first year after diagnosis. Understanding PROs after a breast cancer diagnosis and throughout the first year of survivorship, and the predictors of PROs, is important to help facilitate the transition to breast cancer survivorship.^6^

Physical activity is one lifestyle behavior that may influence QoL and other important PROs including fatigue. One recent network meta-analysis of 45 studies found that aerobic, resistance, and combined aerobic and resistance activity were significantly associated with QoL.^7^ For fatigue, an overview of 29 systematic reviews concluded physical activity interventions to be effective in alleviating cancer-related fatigue in breast cancer survivors.^8^ Few studies have implemented prospective cohort designs to examine how changes in physical activity from diagnosis to posttreatment are associated with changes in PROs. Prospective cohorts allow the opportunity to study physical activity and PROs across a longer period, and in a naturally occurring environment.^9-11^ More studies are now using accelerometers to monitor daily activity patterns among cancer survivors.^12^ Accelerometry provides a more precise, detailed, and reliable measurement across the movement continuum (eg, light, moderate, vigorous-intensity, steps, sedentary time). Implementing self-report physical activity to supplement device-based measures allows for a more complete picture of physical activity volume and expands our understanding of physical activity behaviors beyond the timeframes assessed by device-based measures (ie, prediagnosis and during treatment).

The Alberta Moving Beyond Breast Cancer (AMBER) study is the first and only prospective cohort study designed to examine the role of device-based and self-reported physical activity, sedentary behavior, and health-related fitness in breast cancer survivorship from the time of diagnosis and into survivorship.^13^^,^^14^ We recently examined cross-sectional associations between physical activity, sedentary behavior, and PROs (ie, QoL, fatigue, and depression) shortly after diagnosis.^15^^,^^16^ Here, we examine prospective associations between changes in physical activity and PROs (ie, QoL and fatigue) from diagnosis to 1 year postdiagnosis.

The primary objective of this study was to examine associations between device-measured physical activity (ie, average daily steps, daily light intensity hours, and moderate-to-vigorous physical activity [MVPA] intensity hours) with changes in QoL and fatigue in the first year after diagnosis. The secondary objective was to examine associations between physical activity changes based on self-reported data from the year prior to breast cancer diagnosis to the year following diagnosis with changes in QoL and fatigue. We hypothesized that those with the greatest improvements in device-based and self-reported activity (ie, quartile [Q] 4) 1 year after diagnosis would demonstrate greater improvements in QoL and fatigue profiles compared with participants who reduced (ie, Q1), and did not change (ie, Q2) their physical activity patterns.

## Methods

### Study design and participant recruitment

We have previously published a description of the AMBER study design and methods^13^ as well as a baseline description of the full cohort that participated in the AMBER study (eg, demographic, clinical, behavioral variables).^14^ In summary, the mean age of participants was 54.9 (10.8) years, 55% had stage II or III disease, and 58.2% received chemotherapy. Mean body mass index (BMI) was 27.5 kg/m^2^. We recruited women with newly diagnosed breast cancer between July 2012 and July 2019. Women living in Edmonton or Calgary, Alberta, Canada were eligible if they had histologically confirmed stage I (≥T1c) to stage IIIc breast cancer, were 18-80 years old, were able to complete questionnaires in English, and were not pregnant at the time of recruitment. We obtained ethics approval through the Health Research Ethics Board of Alberta: Cancer Committee (HREBA.CC-17-0576), and each participant completed a signed consent form.

### Timing of assessments and measurements

Participants completed baseline assessments prior to neoadjuvant therapy or within 90 days of surgery and prior to adjuvant therapy. To include those who may have started adjuvant treatment soon after surgery, participants were eligible for the cohort if they had completed up to 2 cycles of chemotherapy or 10 fractions of radiation therapy. In a subset of women who received neoadjuvant treatment, the goal was to complete baseline assessments before initiating adjuvant chemotherapy but always before the third cycle of chemotherapy. Follow-up assessments were repeated 1 year after the baseline assessment date.

The *Baseline Health Questionnaire* included participants sociodemographic characteristics such as age, marital status, ethnicity, education, income, and employment. The questionnaire also assessed participants’ medical history, comorbidities, medication use history, family history of cancer, lifetime smoking and alcohol use. All questionnaires were completed via pencil and paper at a time suitable to the participant.

*Clinical information* about the participants’ cancer diagnosis was extracted from medical charts by trained study staff. Data extracted included date of diagnosis, tumor stage, grade, histology, surgery type, and treatment(s) received.

*Quality of life* was measured using the SF-36 Version 2 (SF-36v2).^17^^,^^18^ This measure yields 8 health domain scales which are aggregated to form 2 distinct component summary measures: physical component summary score (PCS) and mental component summary score (MCS). These scores represent a summary of the individual’s physical and mental health status. In this paper, we present the PCS and MCS data. Low PCS scores suggest limitations in physical functioning and poor general health, while low MCS scores suggest frequent psychological distress due to emotional problems and poor general health. All health domain scales, and component summaries are scored using a *T*-score metric. Scoring the SF-36v2 involves the application of proprietary algorithms (QualityMetric Incorporated, Lincoln, RI).

*Fatigue* was measured using the Functional Assessment of Chronic Illness Therapy-Fatigue (FACIT-F).^19^ The FACIT-F includes 13 items, such as “I feel fatigued” and “I feel weak all over.” Items are scored on a range from 0 to 52, with higher scores indicating less fatigue. A 3.0-point change on the FACT-F is considered a clinically important difference,^20^ defined as the smallest benefit that is of value to patients.^21^

*Physical activity* was assessed using the ActiGraph GT3X+ accelerometer (ActiGraph, LLC, Pensacola, FL). This ActiGraph device records acceleration using a tri-axial accelerometer. Participants wore the device on their right hip during all waking hours for 7 consecutive days. Light intensity activity, MVPA, and MVPA accumulated in 10-minute bouts were estimated using a hybrid machine learning technique that combined a decision tree and an artificial neural network (R Sojourn package version 1.1.0, Soj3x).^22^ The Soj3x prediction method incorporates a broad range of 30 common daily activities in the neural network to estimate the energy cost of activities, and avoids the use of cut-point based methods that can underestimate MVPA.^23^ This method has also been cross-validated in free-living studies using direct observation^22^ and doubly labeled water.^24^

*Daily steps* were measured using the activPAL device (PAL Technologies, Glasgow, Scotland). Participants were instructed to adhere the activPAL device to the front-midline portion of the thigh with stretch tape that was provided, and they reported their time out of and into bed each day via monitor wear logs. Raw data were initially processed to generate event files using activPAL software (CREA v8.10.12.60). These data were further processed using the R-package *activPAL processing* (v0.4.1) to generate daily summaries over the waking day.^25^ Previous work has suggested that the activPAL yields more accurate step counts compared with the ActiGraph.^26^

*Self-report physical activity* was assessed using the Past Year Total Physical Activity Questionnaire (PYTPAQ).^27^ This questionnaire captures physical activities done in the previous 12 months from the time of questionnaire completion. The PYTPAQ assessed the frequency, duration, and intensity of total recreational, occupational, household, and transit physical activity performed over the previous 12 months prior to diagnosis. At 1 year after diagnosis, the PYTPAQ assessed the same variables since diagnosis which included when the participant was going through breast cancer treatment(s). Self-report physical activity was reported in hours/week, and has demonstrated acceptable reliability and validity for the measurement of past-year physical activity.^27^

### Statistical analysis

Descriptive statistics were used to examine demographic, clinical, and behavioral characteristics of the sample. Analyses included preliminary evaluations of the relevant data, including checks for sparsity, distributions, and missingness. We handled missing data on covariates via multivariate imputations through chained equations, which includes all correlated covariates in regression models to avoid reducing the sample size.^28^^,^^29^ For missing independent (ie, activity variables) and dependent (PROs) variable data, we used complete case analysis. We computed change scores for all physical activity variables and PROs (ie, Δ change = 1 year − baseline). For each activity variable, quartiles were generated. We used analysis of covariance to compare PRO change scores (dependent variable) across activity change quartiles (independent variable). All models were adjusted for relevant covariates that demonstrated association (*P* < .20) with the relevant dependent variable including PCS (ie, age, income, comorbidity, tumor grade, chemotherapy, % body fat at 1 year, and VO2_peak_ at 1 year), MCS (ie, age, income, comorbidity, and chemotherapy), and fatigue (ie, age, income, comorbidity, smoking, chemotherapy, and % body fat at 1 year). An α of .05 was used as a threshold for determining statistical significance. All models were generated using SPSS version 29.0.2.

## Results

Baseline demographic and clinical characteristics are presented in Table 1.^10^^,^^11^ To summarize, the mean age of the sample was 55.5 years of age (SD = 10.7). Most were White (87.6%) and had an average BMI of 27.5 (SD = 5.6). Most participants were diagnosed with stage II or III (55%) breast cancer, 40.9% had a mastectomy, and 58.2% received chemotherapy. Of the 1528 recruited into the cohort study, we assessed 884 patients in Calgary and 644 in Edmonton. We collected PRO and accelerometer assessments 55 and 50 days after surgery (median), respectively. Of the sample, 117 (7.7%) participants received neoadjuvant treatment. For participants scheduled to receive chemotherapy, 20% started treatment before their baseline accelerometer assessment. For those scheduled to receive radiation, 6.6% started radiation before their baseline accelerometer assessment. We previously reported baseline sensitivity analyses where we excluded participants who had already started treatment before their baseline accelerometer assessment (*n* = 378) and found that the associations between accelerometer variables and PROs were similar to the full sample analysis.^15^

**Table 1.** Demographic and clinical characteristics of the Alberta Moving Beyond Breast Cancer cohort participants at baseline, 2012-2019 (*N* = 1422).^a^

Characteristic	*N*	%	Mean ± SD
**Demographic**			
**Age at diagnosis**			55.5 ± 10.7
**Study location**			
** Edmonton**	619	43.5	
** Calgary**	803	56.5	
**Marital status**			
** Married or common-law**	1065	74.9	
** Widowed/separated/divorced**	257	18.1	
** Single/never married**	100	7	
**Ethnicity**			
** Caucasian**	1246	87.6	
** Asian**	97	6.8	
** Indian/South Asian**	31	2.2	
** Black**	9	0.6	
** Latino/Hispanic**	18	1.3	
** First Nations/Indigenous/Metis**	13	0.9	
** Other**	8	0.6	
**Education**			
** High school or below**	316	22.2	
** College**	458	32.2	
** University**	373	26.2	
** Graduate school**	275	19.3	
**Annual family income**			
** <$50 000**	227	16	
** 50-100k**	454	31.9	
** 100-150k**	335	23.6	
** >150k**	406	28.6	
**Employment Status**			
** Works <35 hours per week**	950	66.8	
** Works *≥*35 hours per week**	472	33.2	
**Clinical**			
**Body mass index (kg/m^2^)**			27.5 ± 5.6
**Waist circumference (cm)**			92.8 ± 13.4
**Waist-to-hip ratio (cm)**			0.9 ± 0.1
**% body fat**			43 ± 7.2
**Total caloric intake (kcal/day)**			1716 ± 745
**Number of first-degree relative breast cancer family history**			0.3 ± 0.6
**Stage**			
** I**	641	45.1	
** II**	657	46.2	
** III**	124	8.7	
**Histology**			
** Ductal carcinoma**	1203	84.6	
** Invasive ductal and lobular carcinoma mixed**	56	3.9	
** Invasive lobular carcinoma**	150	10.6	
** Other**	13	0.9	
**Mastectomy**			
** Yes**	581	40.9	
** No**	841	59.1	
**Received neoadjuvant therapy**	117	7.7	
**Comorbidity score (0-8)**			0.9 ± 1.0
**Smoking**			
** Never smoker**	820	57.7	
** Past smoker**	511	35.9	
** Occasional smoker**	11	0.8	
** Current smoker**	80	5.6	

a Data are presented as the mean (SD) for continuous variables and frequency (percentage) for categorical variables.

At baseline, 1442 participants had complete (ie, at least 4 valid days) ActiGraph data, 1424 had complete activPAL data (1422 had both ActiGraph and activPAL data at baseline), and 1457 completed the PYTPAQ. At 1 year, 1194 participants had complete ActiGraph data (83% follow-up rate), 1172 had complete activPAL data (82% follow-up rate), and 1276 completed the PYTPAQ (88% follow-up rate). At the 1-year timepoint, there were significantly more participants lost to follow up in Calgary compared to Edmonton for the activPAL and Actigraph assessments (*P* < .05). Reasons for not participating at the 1-year timepoint were consistent across both sites and include deceased, unable to contact, and withdrew (eg, due to health condition, cancer recurrence, work, too busy).

At 1 year, 1104 participants had complete ActiGraph and PRO data and were included in the final models for analysis. For the steps analysis, 1075 participants had complete activPAL and PRO data and were included in the final models for analysis. For self-reported physical activity, 1250 participants had complete PYTPAQ and PRO data and were included in the final models for analysis.

**Figure 1.**
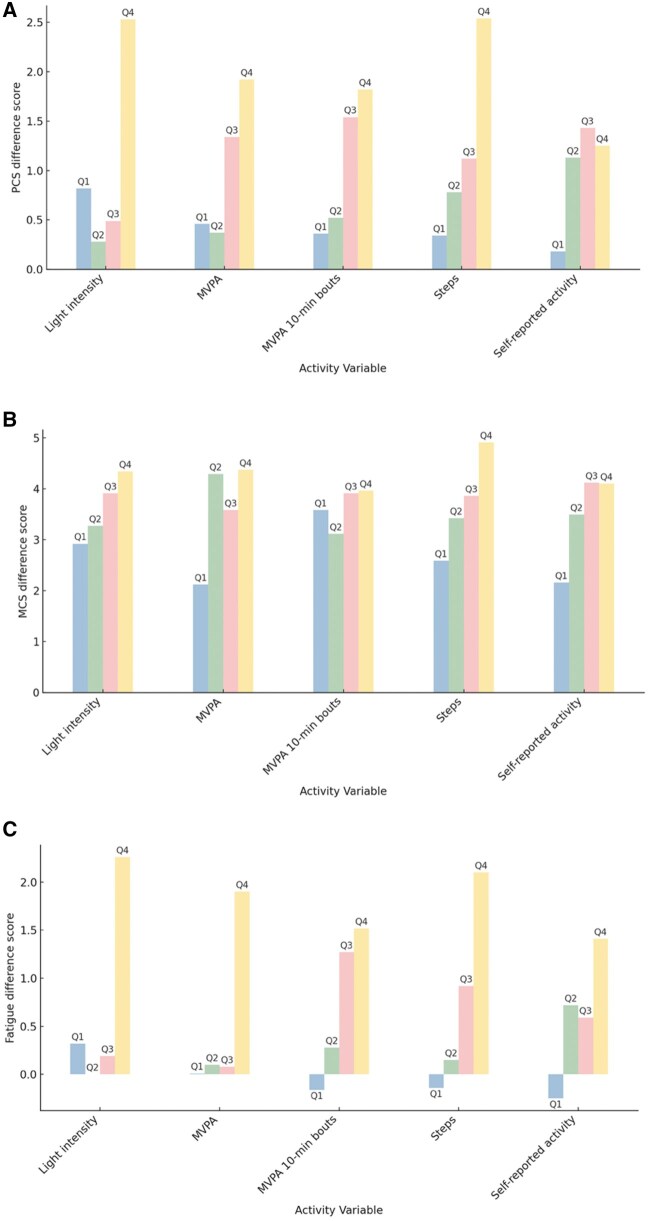
Differences in quality of life and fatigue from baseline to 1 year follow-up across physical activity quartiles in Alberta Moving Beyond Breast Cancer cohort study participants, 2012-2021.

Descriptive information regarding physical activity, daily steps, self-reported physical activity, and PROs both at baseline and at 1 year are included in Table 2. Participants wore the ActiGraph for an average of 5.5 valid days and 14.2 hours each day at baseline, and 5.7 valid days and 14.4 hours at 1 year. Participants wore the activPAL for an average of 5.7 valid days and 14.3 hours at baseline, and 5.8 valid days and 14.4 hours at the 1-year timepoint. All exposure quartiles and means are defined in Tables 3 and 4. In Table 4, we present the mean and median values for Q1 and Q4 given there are no lower and upper bound values to constrain the quartile. Comparison of these values suggests convergence between the mean and median and thus limited influence of values at the tails of the distributions. Table 5 summarizes the differences in changes in PROs across activity variable quartiles, and Figure 1A-C graphically represents the differences in PRO change across activity variable quartiles.

**Table 2.** Baseline and 1 year descriptive statistics for self-reported and device-measured physical activity, quality of life, and fatigue in Alberta Moving Beyond Breast Cancer cohort study participants, 2012-2021.^a^

Variable	**Baseline** ** Mean (SD)**	**1 Year** **Mean (SD)**	***P* value** ^b^
**Device-based physical activity**	*N* = 1442	*N* = 1194	
** Actigraph valid days**	5.51 (1.59)	5.72 (1.44)	<.001
** Actigraph weartime (hours/day)**	14.2 (1.32)	14.4 (1.36)	.003
** Light-intensity activity (hours/day)**	4.51 (1.28)	4.73 (1.33)	<.001
** Moderate-intensity activity (minutes/day)**	52.2 (28.8)	57 (32.4)	<.001
** Vigorous-intensity activity (minutes/day)**	9.6 (11.4)	11.4 (13.2)	<.001
** MVPA (minutes/day)**	61.8 (34.2)	68.4 (38.4)	<.001
** MVPA 10-minute bouts (minutes/day)**	18 (20.4)	19.2 (22.8)	.08
**Daily steps**	*N* = 1424	*N* = 1172	
** activPAL valid days**	5.72 (1.6)	5.8 (1.65)	.088
** activPAL weartime**	14.29 (1.33)	14.41 (1.31)	.006
** Daily steps**	7307 (3018)	7868 (3346)	<.001
**Self-reported physical activity**	*N* = 1457	*N* = 1276	
** Overall physical activity (minutes/day)**	52.1 (19.6)	41.4 (18.6)	
**Health-related quality of life and fatigue**	*N *= 1458	*N* = 1277	<.001
** Physical composite score**	49.24 (7.52)	50.4 (8.2)	<.001
** Mental composite score**	47.84 (10.1)	51.6 (9.1)	<.001
** Fatigue scale (0-52)**	39.25 (9.9)	40.2 (9.7)	.011

Abbreviation: MVPA, moderate and vigorous intensity physical activity.

a Data are presented as the mean (SD).

b Paired-samples *t*-tests were used to test for differences between values.

**Table 3.** Physical activity and step change quartiles in Alberta Moving Beyond Breast Cancer cohort study participants, 2012-2021.

Physical activity outcome	25th percentile	50th percentile	75th percentile	Min to Max	Range
**ActiGraph**					
** Light intensity (minutes/day)**	−30.6	13.2	54.6	−313.2 to 303	616.2
** MVPA (minutes/day)**	−15.6	3.6	24.6	−129 to 340.2	469.2
** MVPA 10-minute bouts (minutes/day)**	−6.6	.00	9	−115.8 to 265.8	381.6
**activPAL**					
** Daily steps**	−1171	289	1796	−18 210 to 11 892	30 102
**PYTPAQ**					
** Self-report physical activity (minutes/day)**	−16.2	−4.7	4.58	−87.6 to 77.00	164.6

Abbreviations: MVPA, moderate and vigorous intensity physical activity; PYTPAQ, Past Year Total Physical Activity Questionnaire.

**Table 4.** Physical activity means (medians) across quartiles in Alberta Moving Beyond Breast Cancer cohort study participants, 2012-2021.^a^

Physical activity outcome	Quartile	Quartile	Quartile	Quartile
	1	2	3	4
**ActiGraph**				
** Light intensity (minutes/day)**	−73.2 (−61.8)	−7.8	32.4	98.4 (86.4)
** MVPA (minutes/day)**	−37.2 (−30.6)	−4.8	13.2	49.8 (40.2)
** MVPA 10-minute bouts (minutes/day)**	−21 (−17.4)	−3	4.2	24 (18)
**activPAL**				
** Daily steps**	−2785 (−2346)	400	975	3811 (3192)
**PYTPAQ**				
** Self-report total physical activity (minutes/day)**	−28 (−25)	−10	0	14 (11)

Abbreviations: MVPA, moderate and vigorous intensity physical activity; PYTPAQ, Past Year Total Physical Activity Questionnaire.

a We present the median value in parentheses for Q1 and Q4 given there are no lower and upper bound values to constrain the quartile.

**Table 5.** Differences in QoL and fatigue change across device-based and self-reported activity (mean and SE) physical activity change quartiles.^a^

	Quartile 1	Quartile 2	Quartile 3	Quartile 4	P value (trend)
	(*N* = 271)	(*N* = 276)	(*N* = 281)	(*N* = 276)	
Light intensity activity** Physical composite score** ^b^	0.82 ± 0.46	0.28 ± 0.46	0.49 ± 0.45	**2.53 ± 0.46** ^1,2v4^	**.002**
** Mental composite score** ^c^	2.92 ± 0.57	3.27 ± 0.57	3.91 ± 0.56	4.34 ± 0.57	.299
** Fatigue** ^d^	0.32 ± 0.56	0.00 ± 0.55	0.19 ± 0.55	**2.26 ± 0.56** ^1,2,3v4^	**.014**
**MVPA**	(*N* = 271)	(*N* = 277)	(*N* = 277)	(*N* = 278)	
** Physical composite score**	0.46 ± 0.47	0.37 ± 0.46	**1.34 ± 0.46** ^2v3^	**1.92 ± 0.46** ^1,2v4^	.053
** Mental composite score**	2.12 ± 0.57	**4.29 ± 0.57** ^1v2^	3.58 ± 0.57	**4.37 ± 0.57** ^1v4^	**.024**
** Fatigue**	0.01 ± 0.56	0.01 ± 0.55	0.08 ± 0.55	**1.9 ± 0.55** ^1,2v4^	**.05**
**MVPA in 10-minute bouts**	(*N* = 280)	(*N* = 309)	(*N* = 238)	(*N* = 277)	
** Physical composite score**	0.36 ± 0.46	0.52 ± 0.44	1.54 ± 0.50	**1.82 ± 0.46** ^1,2v4^	.056
** Mental composite score**	3.58 ± 0.57	3.11 ± 0.54	3.91 ± 0.61	3.96 ± 0.57	.686
** Fatigue**	−0.16 ± 0.55	0.28 ± 0.53	1.27 ± 0.60	**1.52 ± 0.56** ^1v4^	.106
**Daily steps**	(*N* = 265)	(*N* = 269)	(*N* = 272)	(*N* = 268)	
** Physical composite score**	0.34 ± 0.47	0.78 ± 0.47	1.12 ± 0.47	**2.54 ± 0.48** ^1,2,3v4^	**.007**
** Mental composite score**	2.59 ± 0.59	3.42 ± 0.58	3.86 ± 0.58	**4.91 ± 0.58** ^1v4^	**.007**
** Fatigue**	−0.14 ± 0.57	0.15 ± 0.57	0.92 ± 0.56	**2.1 ± 0.57** ^1,2v4^	**.009**
**Self-reported activity**	(*N* = 309)	(*N* = 312)	(*N* = 315)	(*N* = 314)	
** Physical composite score**	0.18 ± 0.45	1.13 ± 0.45	**1.43 ± 0.44** ^1v3^	1.25 ± 0.44	.206
** Mental composite score**	2.16 ± 0.55	3.49 ± 0.54	**4.12 ± 0.53** ^1v3^	**4.1 ± 0.54** ^1v4^	**.039**
** Fatigue**	−0.25 ± 0.53	0.72 ± 0.52	0.59 ± 0.52	**1.41 ± 0.52** ^1v4^	.177

Abbreviation: MVPA, moderate and vigorous intensity physical activity.

a Bold values indicate a significant difference between quartiles (superscript numbers denote quartile groups comparison).

b *Physical composite models* were adjusted for age, income, comorbidity, tumor grade, chemotherapy, % body fat at 1 year, and VO2_peak_ at 1 year.

c *Mental composite models* were adjusted for age, income, comorbidity, and chemotherapy.

d *Fatigue models* were adjusted for age, income, comorbidity, smoking, chemotherapy, and % body fat at 1 year.

### Moderate-to-vigorous physical activity

Participants were categorized into Q1 (decreased MVPA: mean changes = −37.2 minutes/day), Q2 (stable MVPA: mean change = −4.8 minutes/day), Q (modest increase in MVPA: mean change = 13.2 minutes/day), and Q4 (large increase in MVPA: mean change = +49.8 minutes/day). For MVPA, participants in Q4 reported significantly greater improvements in PCS, MCS, and fatigue compared to those in the lower quartiles. Participants in Q4 had the largest improvements in MVPA (a minimum improvement of 25 min/day) and reported significantly greater improvements in PCS compared with those who decreased (Q1: Δ = 1.5, *P* = .026) and those who reported no changes in MVPA (Q2: Δ = 1.6, *P* = .017). Participants with the largest improvements in MVPA also had significantly greater improvements in MCS compared with those who decreased their MVPA (Q1: Δ = 2.2, *P* = .007). Significant differences also emerged on fatigue change scores when comparing those with the largest improvements in MVPA to those participants who decreased their minutes of MVPA (Q1: Δ = 1.9, *P* = .017) and demonstrated no change/maintenance in MVPA (Q2: Δ = 1.9, *P* = .016). Participants in the highest quartile of change in MVPA in 10-minute bouts (Q4: a minimum improvement of 9 minutes/day) reported significantly larger improvements in PCS compared with those who decreased (Q1: Δ = 1.5, *P* = .024; Q2: Δ = 1.3, *P* = .042). A similar pattern emerged for fatigue where those with the largest increases in MVPA in 10-minute bouts reported significantly larger improvements in fatigue compared with those who decreased the most (Δ = 1.7, *P* = .032).

### Light intensity activity

Participants were categorized into Q1 (decreased light intensity: mean change = −73.2 minutes/day), Q2 (stable light activity: mean change = −7.8 minutes/day), Q3 (modest increase in light activity: mean change = +32.4 minutes/day), and Q4 (large increase in light activity: mean change = +98.4 minutes/day). For light intensity activity, participants in Q4 reported significantly greater improvements in PCS and fatigue compared to those in Q1. Participants in Q4 had the largest improvements in light intensity physical activity (ie, a minimum improvement of at least 55 minutes/day) and reported significantly larger improvements in PCS compared with who decreased in minutes/day (Q1: Δ = 1.7, *P* = .009), those who had slight decreases or remained stable (Q2: Δ = 2.2, *P* <.001), and those who had slight improvements (Q3: Δ = 2.0, *P* = .002). Participants with the largest increases in light intensity activity also reported significantly greater improvements in fatigue compared with those in Q1 (Δ = 1.9, *P* = .015), Q2 (Δ = 2.3, *P* = .004), and Q3 (Δ = 2.1, *P* = .008). There were no significant differences in MCS change across light intensity activity quartiles.

### Daily steps

Participants were categorized into Q1 (decreased steps: mean change = −2785 steps/day), Q2 (stable steps: mean change = +400 steps/day), Q3 (modest increase in steps: mean change = +975 steps/day), and Q4 (large increase in steps: mean change = +3811 steps/day). For daily steps, participants in Q4 reported significantly greater improvements in PCS, MCS, and fatigue compared to those in the lower quartiles. Participants in Q4 demonstrated the largest improvements in daily steps (ie, a minimum improvement of 1796 steps per day) and reported significantly larger improvements in PCS compared with participants who demonstrated the largest daily step decreases per day (Q1: Δ = 2.2, *P* = .001), slight step decreases or maintained daily steps (Q2: Δ = 1.8, *P* = .008), and slight improvements in daily steps (Q3: Δ = 1.4, *P* = .033). Participants with the largest increases in daily steps also reported significantly greater improvements in MCS compared with those with the largest decreases (Q1: Δ = 2.3, *P* = .006). For fatigue, participants with the largest step increases also reported significantly larger improvements in fatigue compared with Q1 (Δ = 2.3, *P* = .006) and Q2 (Δ = 2.0, *P* = .015).

### Self-reported past year total physical activity

Participants were categorized into Q1 (decreased total physical activity: mean change = −28 minutes/day), Q2 (small decrease in total activity: mean change = −10 minutes/day), Q3 (no change in total activity: mean change = 0 minutes/day), and Q4 (large increase in total activity: mean change = +14 minutes/day). For self-reported activity, participants in Q4 reported significantly greater improvements in MCS and fatigue compared to those in the lower quartiles. Participants in Q3 (maintained their self-reported physical activity) reported significantly larger improvements in PCS compared with those with the largest decreases in self-reported physical activity (Q1: Δ = 1.3, *P* = .049). For MCS, compared with participants with the largest decreases, significantly greater improvements were reported by those who maintained their activity (Q3: Δ = 2.0, *P* = .011) and those with the largest increases in activity (Q4: Δ = 1.9, *P* = .012). Participants in Q4 reported significantly greater improvements in fatigue compared with Q1 (Δ = 1.7, *P* = .027).

## Discussion

In our sample, participants with the largest increases in physical activity (most often Q4) reported significantly larger improvements in QoL and fatigue outcomes compared with those who demonstrated the largest decreases in physical activity (most often Q1 and Q2). Compared with those who decreased, participants with the largest increases in MVPA, light activity, and daily steps reported significantly greater improvements in PCS, MCS, and fatigue. Participants with the largest increases in MVPA reported changes in QoL and fatigue from diagnosis to 1 year that approached thresholds for determining a minimally important difference in PCS and MCS (3-4 points),^18^^,^^30^ and fatigue (3 points),^31^ defined as the smallest benefit that is of value to patients.^21^

After 1 year, participants were engaging in almost 7 more minutes of MVPA per day (ie, 49.8 minutes per week), and 560 more steps per day compared with their baseline assessment at diagnosis. Research has suggested that women temporarily reduce their self-reported physical activity soon after a breast cancer diagnosis (∼6-8 months postdiagnosis), after which improvements in physical activity are observed after the completion of treatment(s). In a recent prospective study of 1696 newly diagnosed breast cancer patients, women were assessed on physical activity at diagnosis and approximately 8 months postdiagnosis.^32^ A decrease of 1.28 hours was observed at the 8-month timepoint compared with baseline. In the Pathways Study, data from 3000 breast cancer survivors suggested that physical activity reductions were observed at 6 months after baseline, with improvements observed 24 months after baseline.^33^ In a smaller study by Devoogdt et al,^34^ physical activity levels 1 year after surgery did not fully recover. In these studies, physical activity was assessed via self-reported measures which may limit the precision of the activity assessment. We did not include a device-based activity assessment at 6 months to capture a more proximal impact of breast cancer treatment(s) on activity behaviors. Our study does provide robust device-based evidence that women experience a gradual physical activity increase during the months following treatment completion. We may have captured lower than usual baseline levels of physical activity since the participants were recovering from surgery and some had already started adjuvant therapy. Future research could include multiple device-based assessments during treatment-related time periods in the first year postdiagnosis to examine temporal patterns of activity more completely.^35^ Future research should also continue to include self-reported physical activity assessments before diagnosis, and during and after treatment.

Our device-based physical activity assessments only captured a 7-day period of physical activity and did not yield an assessment of physical activity throughout the year after diagnosis. In contrast, we examined a self-reported assessment of past year total physical activity to capture average daily activity minutes in the year before and after diagnosis. This assessment was able to capture the impact of treatment(s) on activity behavior given participants reported engaging in an average of 11 fewer minutes per day (∼20% decrease) in the year after diagnosis compared with the year before diagnosis. Our findings are similar to previously published research. For example, Irwin et al^36^ found that breast cancer survivors reported an 11% decrease (17 minutes per day) in total physical activity between prediagnosis and postdiagnosis timepoints. Our results also corroborate previous research that also reported decreases in physical activity during treatment(s).^32^^,^^33^ The self-reported past year physical activity assessment complements the device-based assessments and provides an assessment of the total physical activity patterns of participants throughout the year after diagnosis.

In our study, participants with the largest increases in MVPA and MVPA accumulated in 10-minute bouts (ie, more intentional movement synonymous with planned and structured physical activity) reported the greatest improvements in QoL and fatigue (Table 4). Participants with the highest MVPA change reported an almost 4.5-point improvement in mental health (ie, MCS), more than double the improvement observed for PCS. The improvement in MCS was 2.2 points higher than the improvement observed in Q1. The notable improvement in MCS across the sample is likely attributed to participants completing their baseline QoL assessment shortly after receiving their diagnosis and having surgery when mental health may have been compromised, and then again 1 year later when participants had likely completed treatments and experienced some relief.

For both MVPA and MVPA in 10-minute bouts, participants in the highest quartiles also reported significantly greater improvements in PCS and fatigue compared with those in the lowest quartile. Participants in the highest quartile of daily step change reported significantly greater improvements in QoL and fatigue suggesting that the overall volume of daily steps (irrespective of length or cadence) is most important for QoL and fatigue benefits. These findings are noteworthy given that fatigue is often reported as the most common and debilitating symptom for breast cancer survivors. The most recent review of fatigue among breast cancer survivors identified 104 studies where 66% of survivors had reported some degree of fatigue, with up to 30% indicating that their fatigue was problematic.^37^

Few prospective cohort studies have explored changes in physical activity and associations with QoL or fatigue from the time of breast cancer diagnosis and throughout treatment.^9-11^ Variability in QoL, fatigue, and physical activity assessment methods (ie, different QoL measurement scales, self-report vs device-based physical activity) make it difficult to compare our research to the few studies that have been previously published. Most similar to our study design, Lucas et al^10^ followed 554 breast cancer survivors in the Women’s Hormonal Initiation and Persistence study. Women were assessed at diagnosis and 1 year after diagnosis with self-reported activity and QoL assessments. Results suggested that changes in activity were not significantly associated with changes in QoL at the follow-up timepoint. Our results suggest that activity behaviors were associated with changes in QoL and fatigue. Our self-reported past year total physical activity data may be more comparable in design to Lucas et al. Although differences across quartiles were small, participants in Q3 and Q4 reported significantly greater improvements in QoL and fatigue compared with those in Q1.

There are strengths and limitations of our study that warrant mention. Key strengths are the use of comprehensive physical activity assessments including both self-report and device-based measurements, and the recruitment of a homogeneous sample of women newly diagnosed with breast cancer. The ActiGraph and activPAL devices are precise and valid tools for measuring activity patterns throughout the day. The activPAL is considered the *gold standard* for the measurement of stepping activity in chronic disease populations.^38^ Our study used the Soj3x processing approach^22^ which differs from the approaches of other studies that have used cut-points to determine time spent in different physical activity intensities. The Soj3x is more sophisticated when compared to cut points as it uses neural network prediction from 30 different activities varying in intensity.^22^ Few studies have examined physical activity and PROs following diagnosis but before initiating adjuvant therapy or neoadjuvant systemic treatments and evaluated again at 1 year after diagnosis. The time after a diagnosis is a phase in the cancer trajectory that is associated with distinct psychosocial needs and women often report psychosocial distress, including anxiety and fear regarding upcoming treatments.^39^ This study has the largest sample of women with breast cancer in the literature to date, and we were able to achieve strong follow-up rates at the 1-year timepoint (ActiGraph = 83%; activPAL = 82%).

A limitation of our study is our inability to capture the acute effects of different treatments and combinations given the 1 year follow-up assessment. While the SF-36 and activity device measurements were both administered soon after diagnosis, it is possible the measurement timeframes do not overlap given the SF-36 items ask participants to recall the past 4 weeks, whereas the devices captured physical activity over the 7 days participants wore them. Given physical activity and PROs were measured concurrently, it is difficult to establish causality. Other limitations include potential sampling bias, as the study population may represent a healthier subset of breast cancer survivors living in Alberta, Canada.

Participants with the largest increases in physical activity had significantly greater improvements in QoL and fatigue 1 year after breast cancer diagnosis compared with those participants that decreased in their physical activity behaviors. To our knowledge, this investigation is one of the first prospective cohort studies of newly diagnosed women with breast cancer to assess device-based physical activity outcomes at diagnosis and 1 year later. These results will help to inform clinical practice and policies about incorporating physical activity as part of adjuvant therapy for newly diagnosed breast cancer, both during and after treatment(s) for improving QoL and reducing fatigue.

## Data Availability

Deidentified data from this study are not available in a public archive, and are not available upon request from the authors.

## References

[1] Bray F , Laversanne M, Sung H, et al Global cancer statistics 2022: GLOBOCAN estimates of incidence and mortality worldwide for 36 cancers in 185 countries. CA Cancer J Clin. 2024;74:229-263. 10.3322/caac.21834 38572751

[2] Mokhatri-Hesari P , Montazeri A. Health-related quality of life in breast cancer patients: review of reviews from 2008 to 2018. Health Qual Life Outcomes. 2020;18:1-25. 10.1186/s12955-020-01591-x 33046106 PMC7552560

[3] Modi ND , Danell NO, Perry RNA, et al Patient-reported outcomes predict survival and adverse events following anticancer treatment initiation in advanced HER2-positive breast cancer. ESMO Open. 2022;7:100475. 10.1016/j.esmoop.2022.100475 35490579 PMC9271483

[4] Modi N , Abuhelwa AY, Rowland A, et al Association between patient-reported outcomes and therapeutic outcomes in patients with breast cancer: a pooled individual-participant data analysis. J Clin Oncol. 2023;41:530. 10.1200/JCO.2023.41.16_suppl.530

[5] Fortin J , Leblanc M, Elgbeili G, Cordova MJ, Marin MF, Brunet A. The mental health impacts of receiving a breast cancer diagnosis: a meta-analysis. Br J Cancer. 2021;125:1582-1592. 10.1038/s41416-021-01542-3 34482373 PMC8608836

[6] Biparva AJ , Raoofi S, Rafiei S, et al Global quality of life in breast cancer: systematic review and meta-analysis. BMJ Support Palliat Care. 2023;13:e528-e536. 10.1136/bmjspcare-2022-003642 PMC10850719 35710706

[7] Li L , Wang Y, Cai M, Fan T. Effect of different exercise types on quality of life in patients with breast cancer: a network meta-analysis of randomized controlled trials. Breast. 2024;78:103798. 10.1016/j.breast.2024.103798 39243565 PMC11408868

[8] Zhou HJ , Wang T, Xu YZ, et al Effects of exercise interventions on cancer-related fatigue in breast cancer patients: an overview of systematic reviews. Support Care Cancer. 2022;30:10421-10440. 10.1007/s00520-022-07389-5 36326908 PMC9715478

[9] Manneville F , Rotonda C, Conroy T, Bonnetain F, Guillemin F, Omorou AY. The impact of physical activity on fatigue and quality of life during and after adjuvant treatment for breast cancer. Cancer. 2018;124:797-806. 10.1002/cncr.31108 29116645

[10] Lucas AR , Kim Y, Lanoye A, et al Longitudinal associations among physical activity and sitting with endocrine symptoms and quality of life in breast cancer survivors: a latent growth curve analysis. Cancer Med. 2023;12:20094-20105. 10.1002/cam4.6581 37768040 PMC10587924

[11] Lope V , Guerrero-ZotanoÁ, Fernández de Larrea-BazN, et al Cross-sectional and longitudinal associations of adherence to WCRF/AICR cancer prevention recommendations with health-related quality of life in breast cancer survivors. Health-EpiGEICAM study. J Nutr Health Aging. 2024;28:100312. 10.1016/j.jnha.2024.10031238970849

[12] Peddle-McIntyre CJ , Cavalheri V, Boyle T, et al A review of accelerometer-based activity monitoring in cancer survivorship research. Med Sci Sports Exerc. 2018;50:1790-1801. 10.1249/MSS.0000000000001644 29683922

[13] Courneya KS , Vallance JK, Culos-Reed SN, et al The Alberta moving Beyond Breast Cancer (AMBER) cohort study: a prospective study of physical activity and health-related fitness in breast cancer survivors. BMC Cancer. 2012;12:525. 10.1186/1471-2407-12-525 23153358 PMC3534483

[14] Friedenreich CM , Vallance JK, McNeely ML, et al The Alberta moving Beyond Breast Cancer (AMBER) cohort study: baseline description of the full cohort. Cancer Causes Control CCC. 2022;33:441-453. 10.1007/s10552-021-01539-6 35064432 PMC8821077

[15] Vallance JK , Friedenreich CM, Wang Q, et al Associations of device-measured physical activity and sedentary time with quality of life and fatigue in newly diagnosed breast cancer patients: baseline results from the AMBER cohort study. Cancer. 2023;129:296-306. 10.1002/cncr.34531 36367438 PMC10695099

[16] Vallance JK , Friedenreich CM, Wang Q, et al Depression, happiness, and satisfaction with life in women newly diagnosed with breast cancer: associations with device-measured physical activity and sedentary time. Psychooncology. 2023;32:1268-1278. 10.1002/pon.6180 37395625 PMC12336974

[17] Ware J , Kosinski M, Dewey J. How to Score Version Two of the SF-36^®^ Health Survey. QualityMetric Incorporated; 2000.

[18] Maruish M. User’s Manual for the SF-36v2 Health Survey. 3rd ed. QualityMetric Incorporated; 2011.

[19] Cella D , Nowinski CJ. Measuring quality of life in chronic illness: the functional assessment of chronic illness therapy measurement system. Arch Phys Med Rehabil. 2002;83:S10-S17. 10.1053/apmr.2002.36959 12474167

[20] Eton DT , Cella D, Yost KJ, et al A combination of distribution- and anchor-based approaches determined minimally important differences (MIDs) for four endpoints in a breast cancer scale. J Clin Epidemiol. 2004;57:898-910. 10.1016/j.jclinepi.2004.01.012 15504633

[21] McGlothlin AE , Lewis RJ. Minimal clinically important difference: defining what really matters to patients. JAMA. 2014;312:1342-1343. 10.1001/jama.2014.13128 25268441

[22] Lyden K , Keadle SK, Staudenmayer J, Freedson PS. A method to estimate free-living active and sedentary behavior from an accelerometer. Med Sci Sports Exerc. 2014;46:386-397. 10.1249/MSS.0b013e3182a42a2d 23860415 PMC4527685

[23] Matthews CE , Keadle SK, Berrigan D, et al Influence of accelerometer calibration approach on moderate-vigorous physical activity estimates for adults. Med Sci Sports Exerc. 2018;50:2285-2291. 10.1249/MSS.0000000000001691 29933344 PMC6193831

[24] Matthews CE , Kozey Keadle S, Moore SC, et al Measurement of active and sedentary behavior in context of large epidemiologic studies. Med Sci Sports Exerc. 2018;50:266-276. 10.1249/MSS.0000000000001428 28930863 PMC5768470

[25] Tudor-Locke C , Ducharme SW, Aguiar EJ, et al Walking cadence (steps/min) and intensity in 41 to 60-year-old adults: the CADENCE-adults study. Int J Behav Nutr Phys Act. 2020;17:137. 10.1186/s12966-020-01045-z 33168018 PMC7654058

[26] Wu Y , Petterson JL, Bray NW, Kimmerly DS, O’Brien MW. Validity of the activPAL monitor to measure stepping activity and activity intensity: a systematic review. Gait Posture. 2022;97:165-173. 10.1016/j.gaitpost.2022.08.002 35964334

[27] Friedenreich CM , Courneya KS, Neilson HK, et al Reliability and validity of the past year total physical activity questionnaire. Am J Epidemiol. 2006;163:959-970. 10.1093/aje/kwj112 16524954

[28] Little R , Rubin D. Statistical Analysis with Missing Data. 2nd ed. John Wiley & Sons; 2002.

[29] van Buuren S. Flexible Imputation of Missing Data. 2nd ed. CRC Press LLC; 2018.

[30] Ogura K , Yakoub MA, Christ AB, et al What are the minimum clinically important differences in SF-36 scores in patients with orthopaedic oncologic conditions? Clin Orthop Relat Res. 2020;478:2148-2158. 10.1097/CORR.0000000000001341 32568896 PMC7431256

[31] Cella D , Eton DT, Lai JS, Peterman AH, Merkel DE. Combining anchor and distribution-based methods to derive minimal clinically important differences on the functional assessment of cancer therapy (FACT) anemia and fatigue scales. J Pain Symptom Manage. 2002;24:547-561. 10.1016/S0885-3924(02)00529-8 12551804

[32] Kwan ML , Sternfeld B, Ergas IJ, et al Change in physical activity during active treatment in a prospective study of breast cancer survivors. Breast Cancer Res Treat. 2012;131:679-690. 10.1007/s10549-011-1788-4 21953007 PMC3273453

[33] Shi Z , Rundle A, Genkinger JM, et al Distinct trajectories of moderate to vigorous physical activity and sedentary behavior following a breast cancer diagnosis: the pathways study. J Cancer Surviv Res Pract. 2020;14:393-403. 10.1007/s11764-020-00856-0 PMC7955660 32130627

[34] Devoogdt N , Van Kampen M, Geraerts I, et al Physical activity levels after treatment for breast cancer: one-year follow-up. Breast Cancer Res Treat. 2010;123:417-425. 10.1007/s10549-010-0997-6 20582717

[35] Courneya KS , McNeely ML, Booth CM, Friedenreich CM. An integrated framework for the study of exercise across the postdiagnosis cancer continuum. Front Oncol. 2024;14:1432899. 10.3389/fonc.2024.1432899 39376986 PMC11456400

[36] Irwin ML , Crumley D, McTiernan A, et al Physical activity levels before and after a diagnosis of breast cancer: the health, eating, activity, and lifestyle (HEAL) study. Cancer. 2003;97:1746-1757. 10.1002/cncr.11227 12655532 PMC3034406

[37] Ruiz-Casado A , Álvarez-Bustos A, de Pedro CG, Méndez-Otero M, Romero-Elías M. Cancer-related fatigue in breast cancer survivors: a review. Clin Breast Cancer. 2021;21:10-25. 10.1016/j.clbc.2020.07.011 32819836

[38] O’Brien CM , Duda JL, Kitas GD, Veldhuijzen van Zanten JJCS, Metsios GS, Fenton SAM. Measurement of sedentary time and physical activity in rheumatoid arthritis: an ActiGraph and activPAL^TM^ validation study. Rheumatol Int. 2020;40:1509-1518. 10.1007/s00296-020-04608-2 32472303 PMC7371657

[39] Hewitt M , Herdman R, Holland J. Psychosocial Needs of Women with Breast Cancer. National Academies Press; 2004. 25009861

